# Supplementary material for: Characterisation and functional predictions of canine long non-coding RNAs
Source: Sci Rep. 2018 Sep 7;8:13444. doi: 10.1038/s41598-018-31770-2 (PMC6128939; doi:10.1038/s41598-018-31770-2)
Supplement: Supplementary file 1 — Supplementary Information [file 41598_2018_31770_MOESM1_ESM.pdf]

Characterization and functional predictions of canine long non-coding RNAs

Céline Le Béguec[1], Valentin Wucher[1,2], Lætitia Lagoutte[1,3], Edouard Cadieu[1], Nadine Botherel[1], Benoît Hédan[1], Clotilde De Brito[1], Anne-Sophie Guillory[1], Catherine André[1], Thomas Derrien[1,*], and Christophe Hitte[1,*]

* Corresponding authors: hitte@univ-rennes1.fr; tderrien@univ-rennes1.fr

Organ	Dog breed	Total reads	Data origin	Anatomical systems
Adrenal_gland	Bernese Mountain Dog	54,776,586	(Wucher et al. 2017)	Unmapped
Blood	Beagle	25,294,750	(Hoeppner et al. 2014)	Unmapped
Brain	Beagle	39,268,738	(Hoeppner et al. 2014)	Nervous system
Cerebellum	Great Swiss Mountain Dog	44,902,865	(Wucher et al. 2017)	Nervous system
Cortex	Belgian Shepherd	41,319,413	(Wucher et al. 2017)	Nervous system
Gut_colon	Bernese Mountain Dog	52,310,396	(Wucher et al. 2017)	Digestive system
Hair_follicle	Labrador	45,694,722	(Wucher et al. 2017)	Integumental system
Heart	Beagle	24,930,702	(Hoeppner et al. 2014)	Musculoskeletal system
Jejunum	Labrador	50,569,866	(Wucher et al. 2017)	Digestive system
Keratinocyte	Beagle	54,482,221	(Hoeppner et al. 2014)	Integumental system
Kidney	Beagle	27,691,951	(Hoeppner et al. 2014)	Unmapped
Liver	Beagle	25,010,943	(Hoeppner et al. 2014)	Unmapped
Lung	Beagle	22,278,693	(Hoeppner et al. 2014)	Unmapped
Mammary_gland	Great Swiss Mountain Dog	44,349,725	(Wucher et al. 2017)	Integumental system
Mucosal	Labrador	44,195,944	(Wucher et al. 2017)	Integumental system
Muscle	Beagle	24,808,375	(Hoeppner et al. 2014)	Musculoskeletal system

Nasal_planum	Labrador	69,193,538	(Wucher et al. 2017)	Integumental system
Olfactory_bulb	Great Swiss Mountain Dog	45,799,491	(Wucher et al. 2017)	Nervous system
Ovary	Beagle	69,193,538	(Hoeppner et al. 2014)	Unmapped
Pancreas	Belgian Shepherd	47,171,936	(Wucher et al. 2017)	Unmapped
Retina	Border Collie	50,480,134	(Wucher et al. 2017)	Unmapped
Skin	Beagle	52,275,710	(Wucher et al. 2017)	Integumental system
Spinal_cord	Great Swiss Mountain Dog	46,844,306	(Wucher et al. 2017)	Nervous system
Spleen	Belgian Shepherd	49,604,583	(Wucher et al. 2017)	Unmapped
Testis	Beagle	26,743,805	(Hoeppner et al. 2014)	Testis
Thymus	Saluki	51,079,197	(Wucher et al. 2017)	Unmapped

Supplementary Table S1. Description of each tissue type, dog breed, number of total reads, publication reference and anatomical systems.

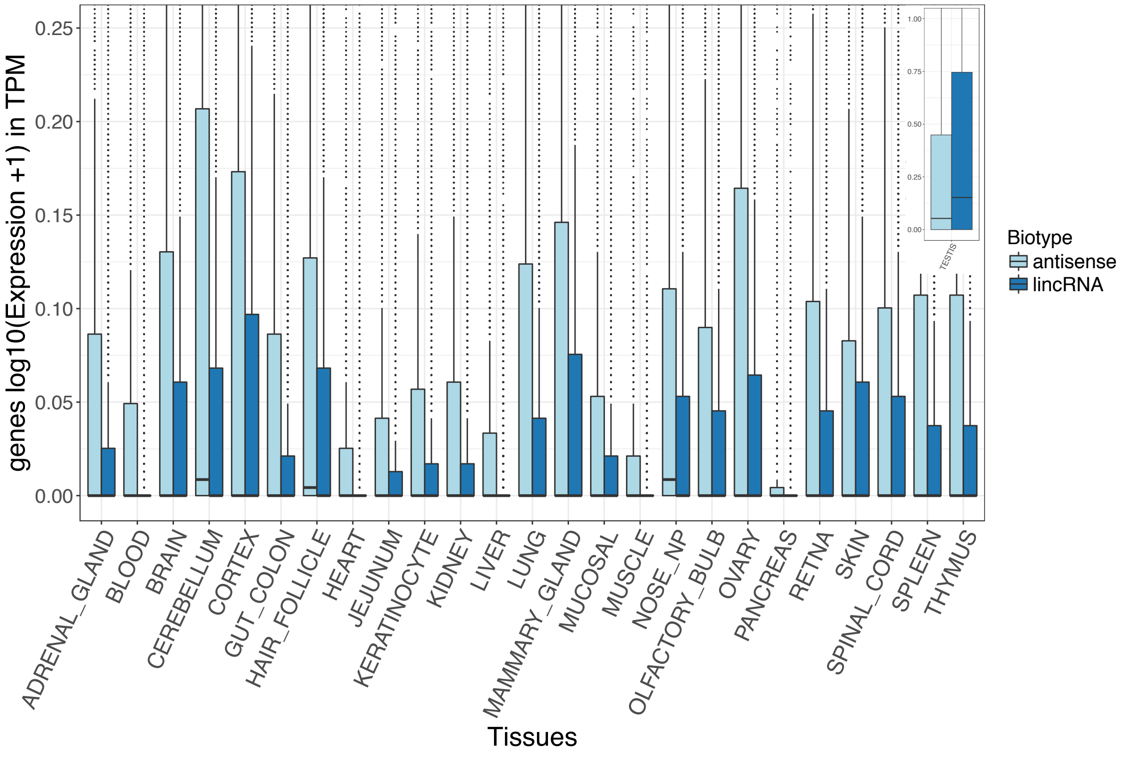

Supplementary Fig. S1. Comparative analysis of log10 transcription (TPM + 1) levels between lncRNAs overlapping an antisense of mRNAs (*antisense*) in light blue and long intergenic non-coding RNAs (lincRNAs) in dark blue genes in 26 canine tissues. Given the high proportion of genes specifically expressed in the testis tissue, it is represented in the box at the top-right of the figure.

Number of gene	%	Tissues	Number of gene	%	Tissues
3,001	65.3	Testis	47	1.0	Keratinocyte
156	3.4	Cerebellum	45	1.0	Blood
138	3.0	Thymus	44	1.0	Olfactory_bulb
132	2.9	Kidney	43	0.9	Spleen
118	2.6	Hair_follicle	40	0.9	Jejunum
104	2.3	Liver	37	0.8	Retina
97	2.1	Ovary	34	0.7	Muscle
90	2.0	Adrenal_gland	34	0.7	Brain
74	1.6	Nasal_planum	32	0.7	Skin
60	1.3	Cortex	31	0.7	Spinal_cord
59	1.3	Heart	30	0.7	Mammary_gland
58	1.3	Lung	24	0.5	Pancreas
53	1.2	Gut_colon	18	0.4	Mucosal

Supplementary Table S2. First column is the number of tissue specific lncRNAs per tissue. Second column is the percentage versus the total number of 4,599 tissue specific lncRNAs. Third column is the tissue.

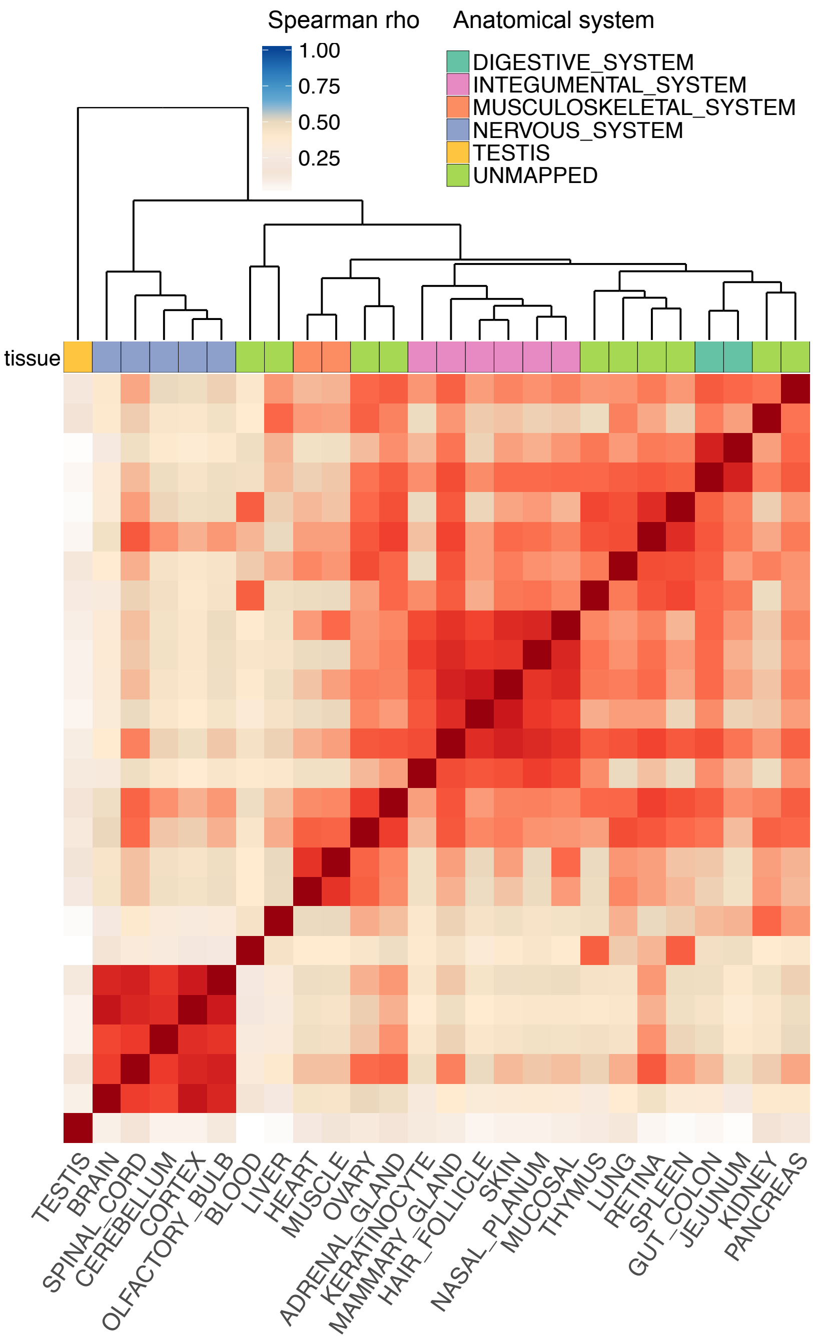

Supplementary Fig. S2. Hierarchical clustering of the 26 canine tissues based on Spearman correlations (Correlation coefficient) measured from protein-coding genes expression data (normalized in TPM).

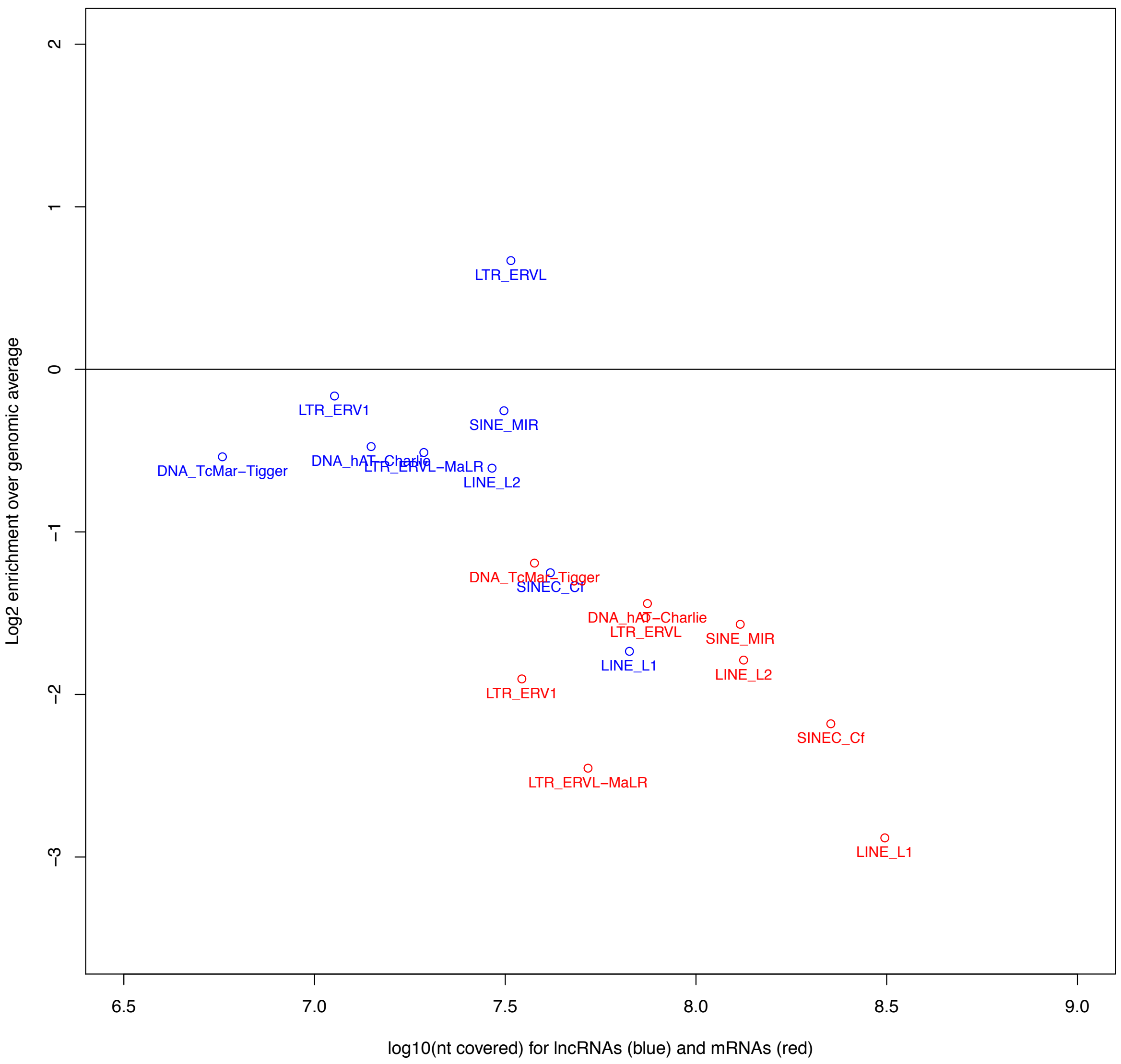

Supplementary Fig. S3. TE family frequencies for lincRNAs (blue) versus mRNAs (red) compared to the genomic average. Larger families are on the right. Enrichments are above zero on the y-axis whereas depletions are below zero.

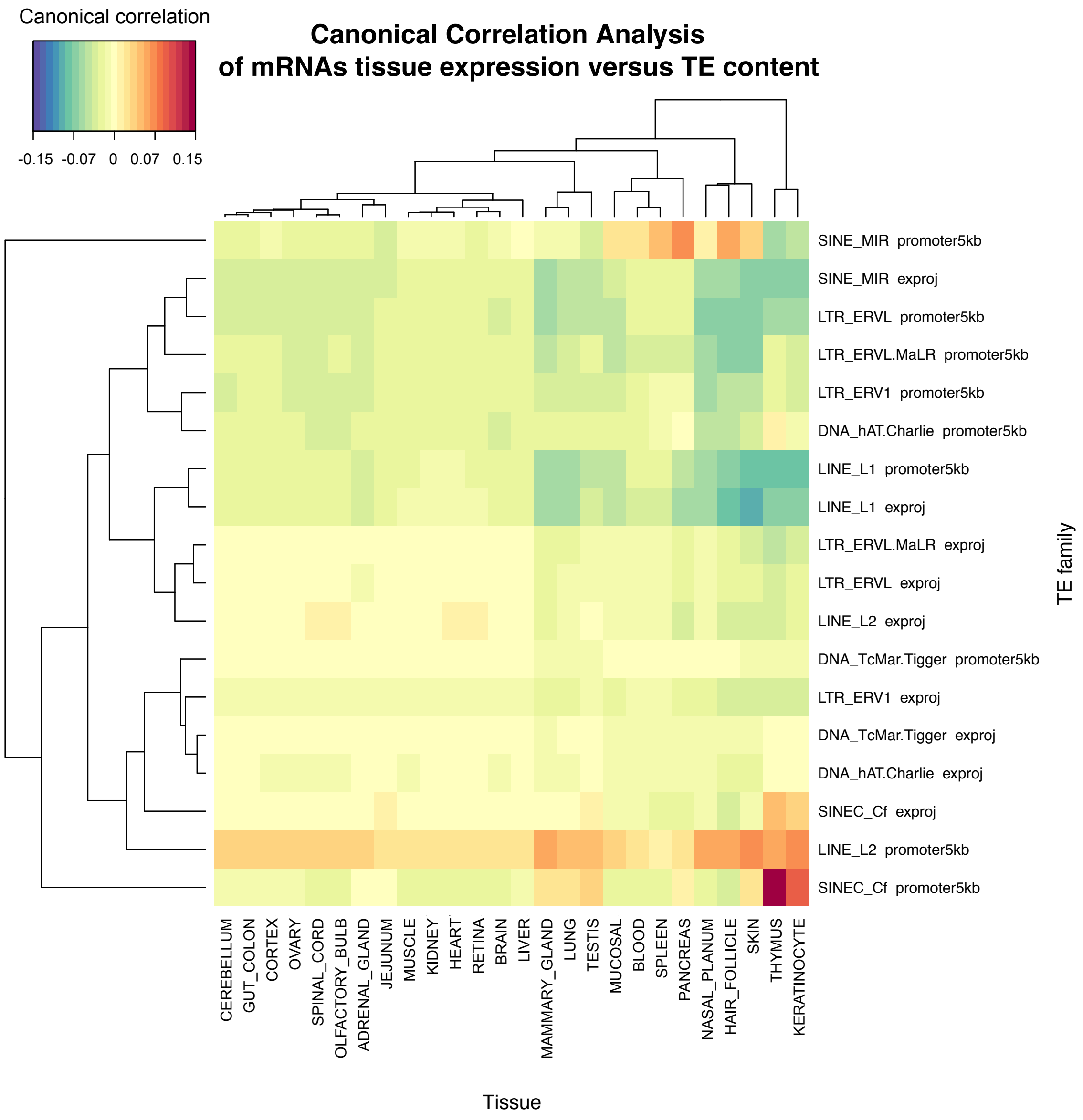

Supplementary Fig. S4. Canonical correlations between mRNAs expression in the 26 tissues (column) and TE families in rows in exons and promoters.

Supplementary Fig. S5. http://dogs.genouest.org/lncRNA.html with all circos (from chromosome 2 to X).

---

		mRNA	lncRNA
Dog	Number of gene	8,832	939
	Mean exon per gene	12.2	3.1
Human	Number of gene	8,832	939
	Mean exon per gene	12.7	3.6

Supplementary Table S3. Effective and mean number of exon per gene (for dog and human data).

Supplementary Fig. S6. http://dogs.genouest.org/lncRNA.html, all bar plots of comparative transcriptomics.

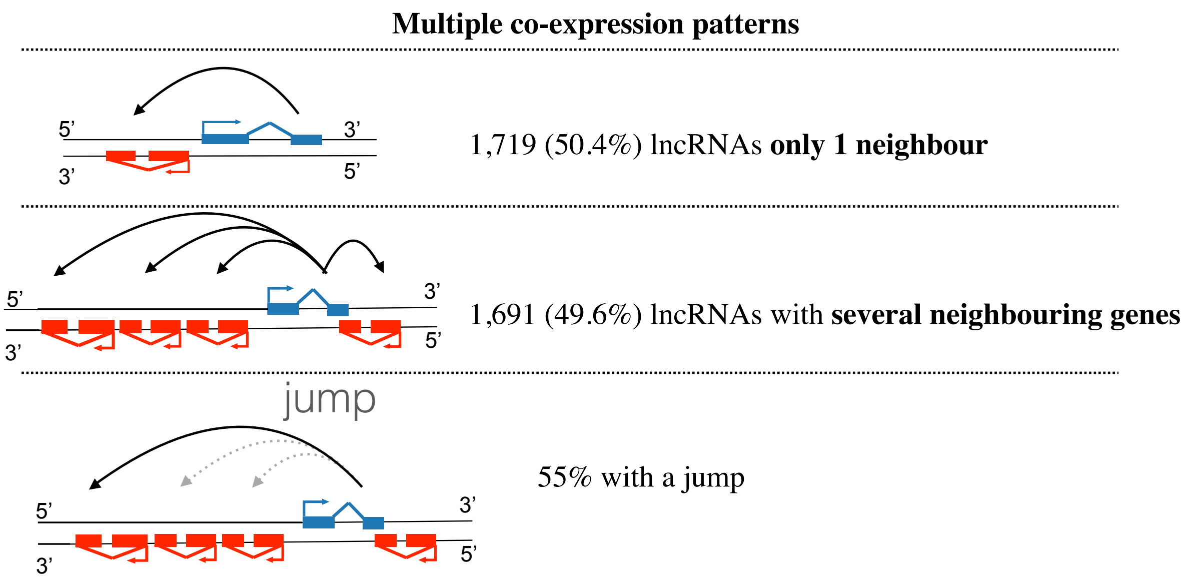

Supplementary Fig. S7. Schema representing multiple co-expression patterns, with only 1 neighbour, several neighbouring genes and a jump.

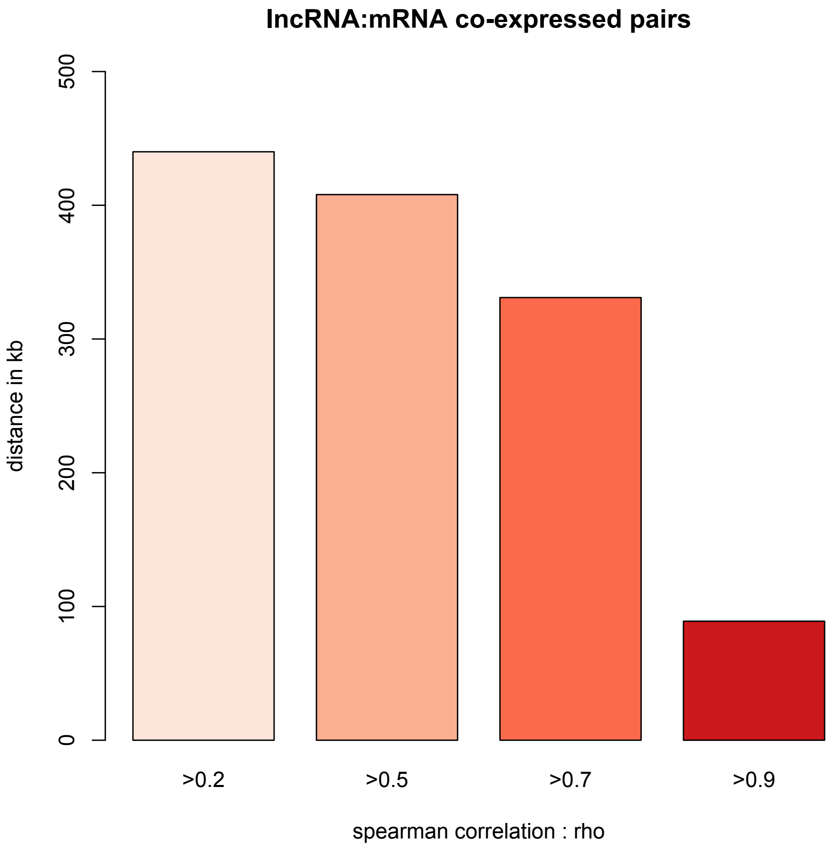

Supplementary Fig. S8. lncRNA:mRNA co-expression Spearman rho values from > 0.2 to > 0.9 as a function of the distance between lncRNA and mRNA.

GO terms	Description	FDR	Nb of genes
GO:0007423	Sensory organ development	0.6^{e-3}	147
GO:0007389	Pattern specification process	0.7^{e-3}	127
GO:0048736	Appendage development	4.1^{e-3}	58
GO:0048089	Positive regulation of cellular component biogenesis	4.1^{e-3}	129
GO:0061564	Axon development	5.1^{e-3}	129
GO:0018205	Peptidyl lysine modification	5.1^{e-3}	115
GO:0051961	Negative regulation of nervous system development	6.7^{e-3}	81
GO:0030902	Hinbrain development	6.7^{e-3}	49
GO:0010975	Regulation of neuron projection development	7.5^{e-3}	116
GO:0048568	Embryonic organ development	8.9^{e-3}	118
GO:0050804	Modulation of synaptic transmission	8.9^{e-3}	84
GO:0016049	Cell growth	1.1^{e-2}	135
GO:0007033	Vacuole organization	1.1^{e-2}	54
GO:0016570	Histone modification	1.1^{e-2}	120
GO:0060021	Palate development	1.2^{e-2}	32
GO:0060560	Developmental growth involved in morphogenesis	1.7^{e-2}	65
GO:0051962	Positive regulation of nervous system development	1.9^{e-2}	121
GO:0001701	In utero embryonic development	2.0^{e-2}	92
GO:0022604	Regulation of cell morphogenesis	2.2^{e-2}	118
GO:0016358	Dendrite development	2.4^{e-2}	58
GO:0007224	Smoothened signaling pathway	3.5^{e-2}	40
GO:0010720	Positive regulation of cell development	4.0^{e-2}	118

Supplementary Table S4. Enrichment analysis of mRNA GO terms significantly co-expressed in 8,139 lncRNA:mRNA pairs using the WebGestalt website (www.webgestalt.org/). GO terms are ranked according to their FDR together with the number of genes presenting the specific GO term.

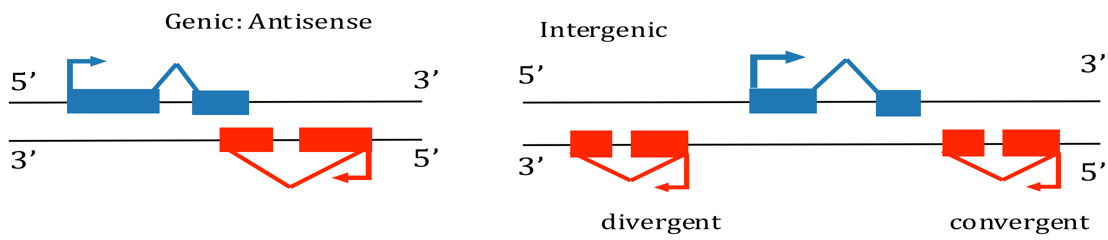

Supplementary Fig. S9. Schema representing *antisense* and *lincRNA* (divergent and convergent).
